# Rice homolog of Sin3‐associated polypeptide 30, OsSFL1, mediates histone deacetylation to regulate flowering time during short days

**DOI:** 10.1111/pbi.13235

**Published:** 2019-09-06

**Authors:** Yuke Geng, Pingxian Zhang, Qing Liu, Ziwei Wei, Adeel Riaz, Sadaruddin Chachar, Xiaofeng Gu

**Affiliations:** ^1^ Biotechnology Research Institute Chinese Academy of Agricultural Science Beijing China

**Keywords:** histone deacetylation, heading date, photoperiodic flowering, HDAC, OsSFL1, rice

The developmental transition from vegetative to reproductive phase (i.e. flowering) is the critical event in plant's life cycle, which is regulated by exogenous and endogenous signals to ensure it in a timely manner (He and Li, 2018). Rice (*Oryza sativa*) is classified as a facultative short‐day plant (Tsuji *et al*., 2011). In rice, *Hd1 (Heading date 1)* is controlled by photoperiod, encodes a zinc finger protein and promotes floral transition under SDs by up‐regulating *Hd3a (Heading date 3a)* expression; while under LDs, it strongly represses *Hd3a* expression to restrain floral transition (Sun *et al*., 2014). Histone acetylation levels are mediated by histone acetyltransferases (HATs) and histone deacetylases (HDACs). HDACs remove acetyl group from histones, leading to a condensed chromatin structure and repressing gene expression, whereas HATs relax chromatin structure and activate gene expression (Liu *et al*., 2014). As one of best characterized HDAC complexes, the Sin3‐HDAC consists of the deacetylase enzyme RPD3, the scaffold Sin3‐HDAC and the structural proteins Sin3‐Associated Polypeptides 18 and 30 (SAP18 and SAP30; Ahringer, 2000). Remarkably, two SAP30‐like proteins in *Arabidopsis*, AFR1and AFR2 have been reported to outline a detailed mechanism in response to light exposure by which histone deacetylation regulates plant flowering at right time (Gu *et al*., 2013). However, the molecular function of SAP30 homolog in rice and whether it could be involved in regulation of flowering time remained unknown. Our study investigated the rice homolog of yeast SAP30 (SAP30 Functional Like 1, OsSFL1; LOC_Os04g08450) causing delayed flowering under SDs.

To investigate biological function of OsSFL1, we first produced *ossfl1* (CRISPR‐Cas9 editing) and *ossfl1‐T* (T‐DNA insertion) lines (Figure 1a). The editing line, *ossfl1*, was identified at the first exon with 28‐bp deletion of *OsSFL1* in Nip background with the target SG sequence (5′‐TCTCCGCCTCGGTACGAGAGTGG‐3′) fused into the engineered CRISPR/Cas vector as reported previously (Feng *et al*., 2013). The *ossfl1‐T* (PFG_3A‐07944.L) line was obtained from the RiceGE database (http://signal.salk.edu/cgi-bin/RiceGE) with Dongjin (DJ) background and identified as a knockout line with insertion at the second intron. In *ossfl1*, we observed a significant delay of almost 2 weeks compared with Nip under SDs (Figure 1b); however, no significant difference was observed under LDs (Figure 1c). Consistently, we also verified that *ossfl1‐T* line delayed flowering time under SDs (about 17 days) compared with that of wild‐type DJ plants (Figure 1d), suggesting that *OsSFL1* is an important player to regulate flowering time in rice.

**Figure 1:**
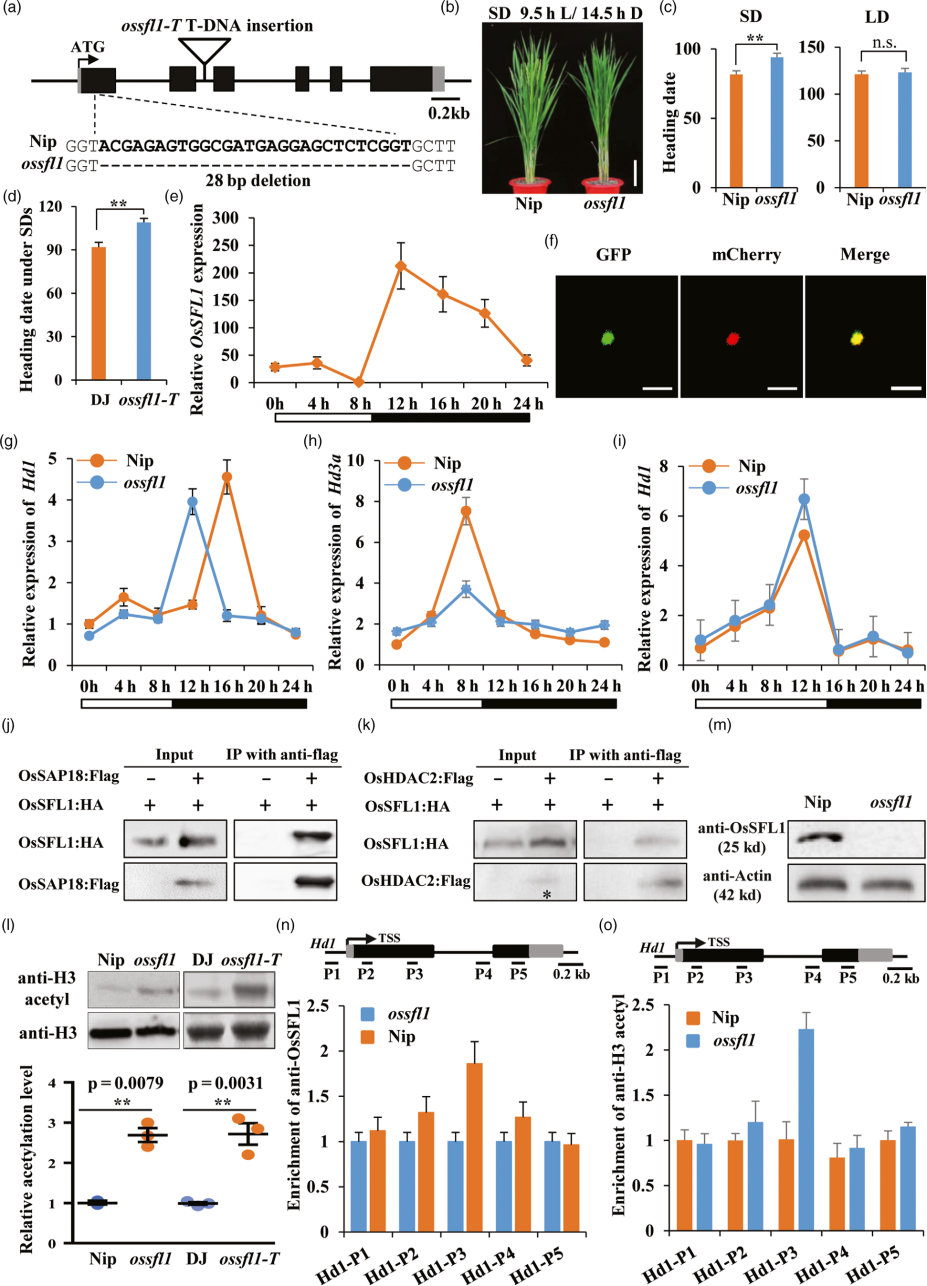
Loss‐of‐function alleles of *ossfl1* delayed flowering time in rice under SDs. (a) Schematic diagram of OsSFL1 mutant lines. The sgRNA mediating the CRISPR–Cas9 target sites of *OsSFL1* was indicated by the dotted line. The T‐DNA insertion mutant *ossfl1‐T* was indicated with hollow triangle. (b) Phenotype of Nip and *ossfl1* lines under SDs (9.5 h light/14.5 h dark). Bar = 10 cm. (c) Heading date of Nip and *ossfl1* lines under SDs and LDs (14.5 h light/9.5 h dark). Bars indicated for standard deviation (s.d.); double asterisks indicated statistically significant differences revealed by two‐tailed Student's *t* test (**, *P* < 0.01). (d) Heading date of DJ and *ossfl1‐T* lines under SDs. (e) Diurnal expression patterns of *OsSFL1* in wild‐type Nip at SDs. *OsActin1* was used as a reference gene. White and dark bars below the *X*‐axis indicate light and dark periods, respectively. (f) Nuclear localization of the OsSFL1‐GFP fusion protein in rice protoplast. OsSFL1‐GFP and mCherry fluorescence were imaged using a laser scanning confocal microscope. The nuclear marker OsMADS3 fused with mCherry was used as a nuclear localization control. Scale bars = 50 μm. (g‐h) Diurnal expression patterns of *Hd1* (g) and *Hd3a* (h) under SDs. (i) Diurnal expression pattern of *Hd1* under LDs. (j‐k) Co‐immunoprecipitation of OsSFL1 with OsSAP18 (j) or OsHDAC2 (k) in rice protoplasts. Total protein extracts from rice protoplasts transformed with OsSFL1:HA and OsSAP18:FLAG or OsHDAC2:FLAG, then fractionated through SDS‐PAGE gel electrophoresis (input) and immunoprecipitated with anti‐FLAG agarose (Sigma, Cat#: A2220). The asterisk indicated appearance of input band. (l) The Western blot results immunoblotted with anti‐H3acetyl (K9 + K14 + K18 + K23 + K27) (Abcam, Cat#: ab47915) in *OsSFL1* mutants and wild types. Band intensities were quantified by the ImageJ program. (m) Verification of synthesized OsSFL1 polyclonal antibody by Western blot (WB) assay (anti‐Actin as a control). (n‐o) Anti‐OsSFL1 (n) and anti‐H3 acetyl at K9 + K14 + K18 + K23 + K27 (o) enrichment at *Hd1* loci under SDs. The amounts of immunoprecipitated genomic fragments were measured by real‐time quantitative PCR and normalized to *OsActin1* as an internal control.

We then investigated diurnal expression levels of *OsSFL1* under SDs. Exposure to light, *OsSFL1* was slightly peaked at midday of SD (zeitgeber time 4, ZT4), and after 2.5 h darkness exposure (ZT12), the transcript levels were increased, but decreased before dawn (Figure 1e). The full‐length CDS of *OsSFL1* was fused with a GFP tag in the PAN580‐GFP vector and then transformed into rice protoplasts of 12‐day‐old etiolated seedlings; this model confirmed that the in‐frame OsSFL1:GFP fusion protein was specific to the nucleus (Figure 1f), indicating that the nuclear protein OsSFL1 displayed diurnal expression pattern.

To determine how *OsSFL1* regulates the functions of photoperiodic genes, the diurnal expression of *Hd1* and *Hd3a* was detected under SDs. The results showed that the expression level of *Hd1* peaked at ZT16 in Nip but peaked at ZT12 in *ossfl1* (Figure 1g). Furthermore, in *ossfl1*,* Hd3a* transcripts under SDs was significantly reduced at ZT8 (Figure 1h). We then detected *Hd1* expression in *ossfl1* under LDs. The diurnal expression of *Hd1* under LDs was not significantly different between Nip and *ossfl1* (Figure 1i). Concomitantly, our results indicated that *OsSFL1* was involved in altering photoperiodic rhythm of *Hd1* under SDs but not under LDs, and then repressed *Hd3a* expression at the end of SDs.

Next, we confirmed that if OsSFL1 could directly interact with other Sin3‐HDAC members, OsSAP18 and OsHDACs. We conducted Co‐IP experiments to check the corresponding interactions by using a transient expression system. The data showed that anti‐FLAG (that recognized OsSAP18:FLAG or OsHDAC2:FLAG) efficiently immunoprecipitated OsSFL1:HA, revealing that OsSFL1 was associated with OsSAP18 (Figure 1j) and OsHDAC2 (Figure 1k), respectively. Collectively, our results suggested that OsSFL1 strongly interacted with OsSAP18 and OsHDAC2 to form OsSFL1‐HDAC complex.

Next, to explore whether mutation of *OsSFL1* could influence global H3 acetylation level, we performed Western blot assays using 4‐week‐old rice seedlings grown under SDs incubated with anti‐H3 acetyl (K9 + K14 + K18 + K23 + K27) antibody. After visualizing by enhanced chemiluminescence (ECL) systems, we found that global H3 acetylation level was significantly increased in both *ossfl1* and *ossfl1‐T* (Figure 1l). Remarkably, with band intensities quantified by the ImageJ software program, we found almost twofold increase in global H3 acetylation level compared to that of wild types (Figure 1l), suggesting that OsSFL1 could mediate histone deacetylation in rice genome.

We further tested whether OsSFL1 could bind to *Hd1* chromatin under SDs to mediate periodic histone deacetylation. Firstly, the synthesized OsSFL1 polyclonal antibody was verified by Western blot analysis using total proteins extracted from Nip and *ossfl1* seedlings (Figure 1m). The ChIP‐qPCR data revealed that OsSFL1 proximally bound to the first exon region of *Hd1* under SDs in 4‐week‐old seedlings of Nip and *ossfl1* (Figure 1n). We then examined H3 acetylation of *Hd1* chromatin using an anti‐H3 acetyl (K9 + K14 + K18 + K23 + K27) antibody. The results showed that loss of OsSFL1 function could lead to an increase in the H3 acetylation level of *Hd1* in the first exon region (Figure 1o), which is consistent with the binding region of OsSFL1. Together, these results concluded that OsSFL1 mediated periodic histone deacetylation on the rhythmically altered photoperiodic gene *Hd1* to specifically dampen the expression of its downstream gene *Hd3a* under SDs.

Our results collectively uncovered a chromatin mechanism of ‘periodic histone deacetylation’ for a day‐length‐dependent regulation of flowering time in rice. Previous studies have reported that several clock‐regulated genes were involved in the alteration of period length and pattern to regulate flowering time in plants (Millar, 2016). Our results showed that the peak shift in *Hd1* expression to an earlier time point might reduce *Hd1* stability in *ossfl1* under SDs (possibly miss‐timing relative to factors that stabilize *Hd1*), and the alteration of diurnal and nocturnal expression pattern of *Hd1* suppress *Hd3a* expression. However, *Arabidopsis* SAP30 homolog proteins (AFRs) have been found repressing *FT* expression in response to inductive LDs and *afr1;afr2* led to precocious flowering (Gu *et al*., 2013), which suggests that SAP30 homologs of *Arabidopsis* and rice have different regulation of photoperiodic pathway. The difference in regulation of flowering time in both species may occur due to different photoperiodic behaviour as rice is a facultative SD plant while *Arabidopsis* is a facultative LD plant (Tsuji *et al*., 2011). Moreover, Hd1 (*Arabidopsis* CO homolog) promotes floral transition through activating *Hd3a* (*Arabidopsis FT* homolog) under SDs and strongly represses floral transition via down‐regulating *Hd3a* under LDs, while CO was only found in promotion of *FT* in *Arabidopsis* under LDs (Tamaki *et al*., 2007). Thus, our results and previous report clearly show the existence of diverged potential molecular mechanism underlying SAP30 homologs in plants.

## Author contributions

X.G. conceived the study and designed the experiments. Y.G. and P.Z. performed the most experiments. Q.L. and Z.W. performed the expression analysis. A.R. and S.C. contributed to the writing. P.Z. and X.G. wrote the manuscript.

## Conflict of interest

The authors declare no conflict of interest.

## References

[pbi13235-bib-0001] Ahringer, J. (2000) NuRD and SIN3: histone deacetylase complexes in development. Trends Genet. 16, 351–356. 10904264 10.1016/s0168-9525(00)02066-7

[pbi13235-bib-0002] Feng, Z. , Zhang, B. , Ding, W. , Liu, X. , Yang, D.L. , Wei, P. , Cao, F. et al. (2013) Efficient genome editing in plants using a CRISPR/Cas system. Cell Res. 23, 1229–1232. 23958582 10.1038/cr.2013.114 PMC3790235

[pbi13235-bib-0003] Gu, X. , Wang, Y. and He, Y. (2013) Photoperiodic regulation of flowering time through periodic histone deacetylation of the florigen gene *FT* . PLoS Biol. 11, e1001649. 24019760 10.1371/journal.pbio.1001649 PMC3760768

[pbi13235-bib-0004] He, Y. and Li, Z. (2018) Epigenetic environmental memories in plants: establishment, maintenance, and reprogramming. Trends Genet. 34, 856–866. 30144941 10.1016/j.tig.2018.07.006

[pbi13235-bib-0005] Liu, X. , Yang, S. , Zhao, M. , Luo, M. , Yu, C.W. , Chen, C.Y. , Tai, R. *et al*. (2014) Transcriptional repression by histone deacetylases in plants. Mol. Plant, 7, 764–772. 24658416 10.1093/mp/ssu033

[pbi13235-bib-0006] Millar, A.J. (2016) The intracellular dynamics of circadian clocks reach for the light of ecology and evolution. Annu. Rev. Plant Biol. 67, 595–618. 26653934 10.1146/annurev-arplant-043014-115619

[pbi13235-bib-0007] Sun, C. , Chen, D. , Fang, J. , Wang, P. , Deng, X. and Chu, C. (2014) Understanding the genetic and epigenetic architecture in complex network of rice flowering pathways. Protein Cell, 5, 889–898. 25103896 10.1007/s13238-014-0068-6 PMC4259885

[pbi13235-bib-0008] Tamaki, S. , Matsuo, S. , Hann, L.W. , Yokoi, S. and Shimamoto, K. (2007) Hd3a protein is a mobile flowering signal in rice. Science, 316, 1033–1036. 17446351 10.1126/science.1141753

[pbi13235-bib-0009] Tsuji, H. , Taoka, K.I. and Shimamoto, K. (2011) Regulation of flowering in rice: two florigen genes, a complex gene network, and natural variation. Curr. Opin. Plant Biol. 14, 45–52. 20864385 10.1016/j.pbi.2010.08.016

